# Supplementary material for: Molecular Viral Diagnosis and Sanitation of Yam Genetic Resources: Implications for Safe Yam Germplasm Exchange
Source: Viruses. 2020 Sep 29;12(10):1101. doi: 10.3390/v12101101 (PMC7650539; doi:10.3390/v12101101)
Supplement: Supplementary file 1 [file viruses-12-01101-s001.zip › viruses-893132-SUP/Figure S2.pdf]

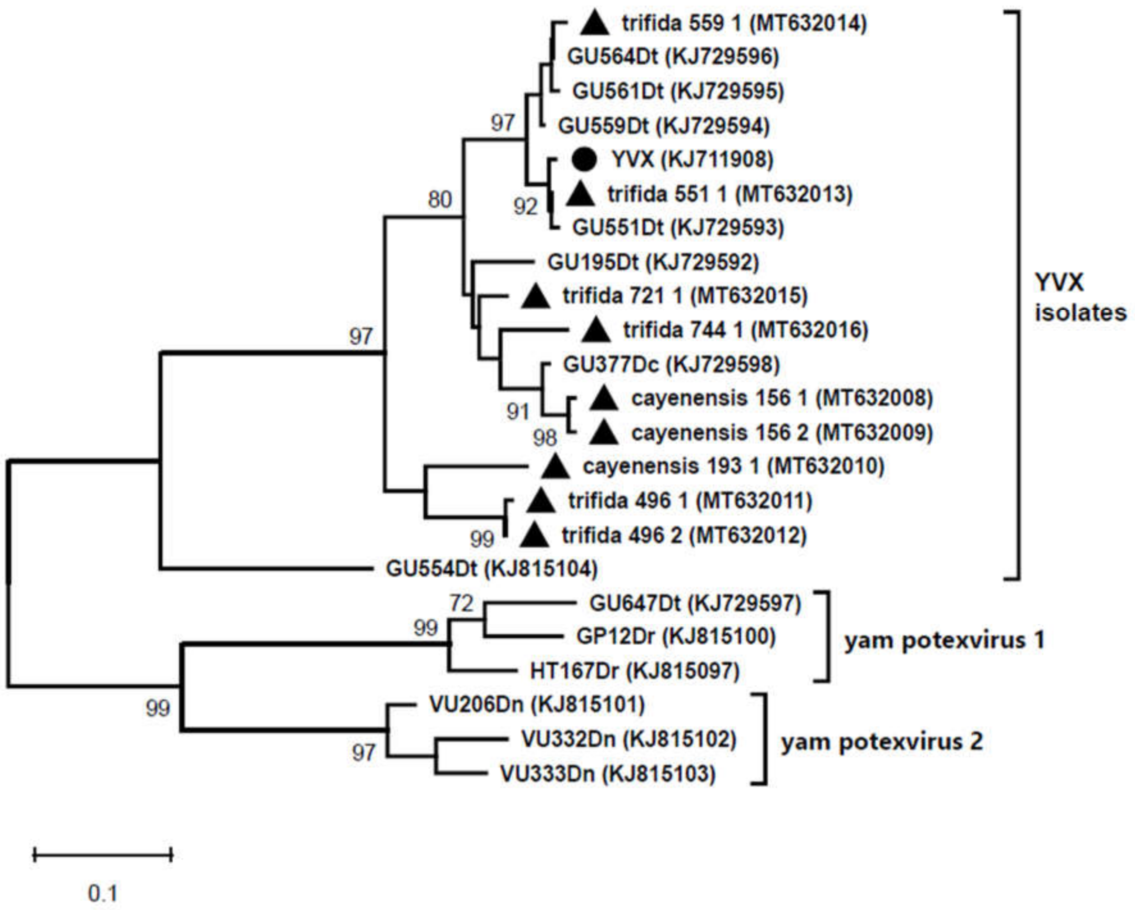

Maximum likelihood phylogenetic tree of partial nucleotide sequences amplified from yam potexviruses using primers YamX-3F and YamX-5R. Previously reported sequences of the same region (208 nt) in the genomes of YVX, yam potexvirus 1 and yam potexvirus 2 were included as references. Evolutionary distances were computed using the HKY model [43]. Bootstrap values of 1,000 replicates are shown at nodes when above 70%. Black triangles represent the sequences generated in this study and the corresponding domain of the YVX complete genome is indicated by a black circle. Accession numbers are provided and the scale bar shows the number of substitutions per base.

43 Hasegawa, M.; Kishino, H.; Yano, T. Dating of the human-ape splitting by a molecular clock of mitochondrial DNA. *J. Mol. Evol.* **1985**, *22*, 160–174.
