# Supplementary figures and images for: Molecular Viral Diagnosis and Sanitation of Yam Genetic Resources: Implications for Safe Yam Germplasm Exchange
Source: Viruses. 2020 Sep 29;12(10):1101. doi: 10.3390/v12101101 (PMC7650539; doi:10.3390/v12101101)

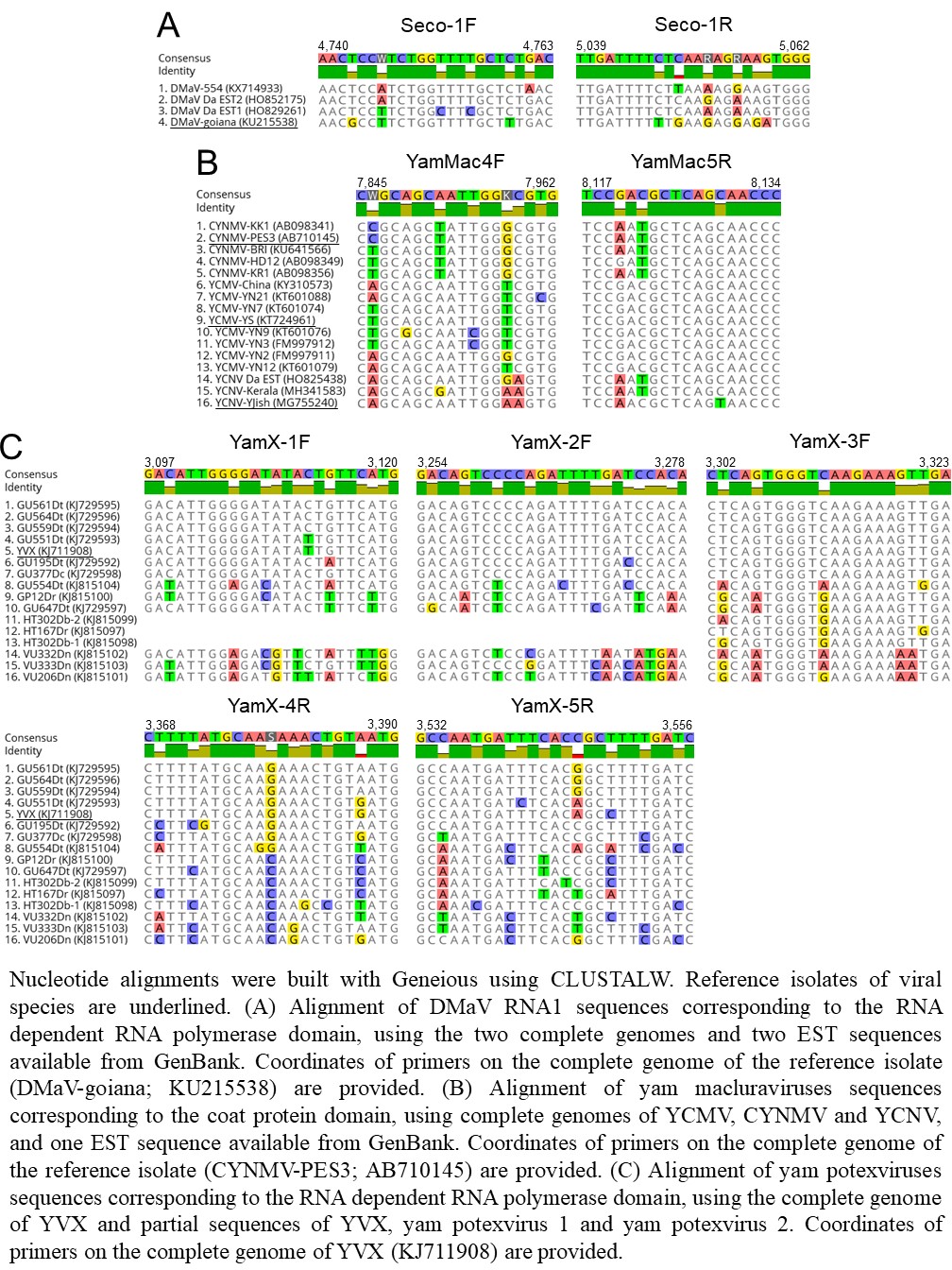

Supplement: Supplementary file 1 [file viruses-12-01101-s001.zip › Figure S1.jpg]
